# Acute Gastroenteritis Induced Adrenal Crisis in a patient with Congenital Adrenal Hyperplasia: A Case Report

**DOI:** 10.31729/jnma.7926

**Published:** 2023-01-31

**Authors:** Nibedita Chapagain, Aayam Adhikari, Nishob Adhikari, Santosh Paudel, Deependra Mandal

**Affiliations:** 1 Kathmandu Medical College and Teaching Hospital, Sinamangal, Kathmandu, Nepal

**Keywords:** *adrenal insufficiency*, *gastroenteritis*, *glucocorticoids*

## Abstract

Congenital Adrenal Hyperplasia is a group of autosomal recessive diseases due to deficiencies of enzymes involved in steroidogenesis. If not diagnosed and treated adequately, Congenital Adrenal Hyperplasia can lead to an acute adrenal crisis with hemodynamic collapse. Acute stressors and steroid insufficiency precipitate an adrenal crisis. The major clinical features are hypotension and volume depletion. Nonspecific symptoms such as fatigue, lack of energy, anorexia, nausea, vomiting, and abdominal pain are common. We report a case of a 3-year male previously diagnosed with congenital adrenal hyperplasia in adrenal crisis secondary to medicine noncompliance and gastroenteritis. The diagnosis was based on the clinical history and biochemical investigations. After initial resuscitation was managed; lifelong oral prednisolone and fludrocortisone were prescribed.

## INTRODUCTION

Congenital Adrenal Hyperplasia (CAH) is a group of disorders encompassing enzyme deficiencies in the adrenal steroidogenesis pathway causing various alterations in glucocorticoid, mineralocorticoid, and sex steroid production.^1^ Adrenal crisis is a life-threatening complication that develops if CAH is not diagnosed early. The predominant manifestation of the adrenal crisis is shocks.^3^ Precipitating factors for adrenal crisis include gastrointestinal diseases, withdrawal of glucocorticoids, and Infections.^4^ Suspected adrenal crisis requires immediate management with hydrocortisone, fluid, and monitoring.^5^ Despite the diagnostic and therapeutic advances adrenal crisis remains a potentially lethal condition.^2^ Here we report a case of adrenal crisis in previously diagnosed CAH patients.

## CASE REPORT

We present a case of a 3 years old male toddler who presented to emergency department with complaints of vomiting and loose stools for 1 day. The child had passed for 4 episodes of non-blood and non-mucus stained stools on that day along with 3 episodes of non-projectile vomiting containing food particles. The patient's mother also complained of multiple episodes of abnormal body movement, each lasting around two minutes in the form of stiffening of limbs, uprolling of eyes, and frothing from the mouth with involuntary passage of urine. The patient is a known case of CAH at 40 days of infancy and was treated with syrup prednisolone and tablet fludrocortisone 0.3 mg/day.

On general examination, the child was ill-looking and delirious. The oral mucosa and tongue were dry with skin turgor returning very slowly. On arrival, the patient had an SPO_2_ of 76% on room air and an RR of 45 per minute. He had subcostal retractions along with diffuse bilateral wheeze on auscultation. Investigations showed deranged electrolytes and leukocytosis with a total count of 20100 and a differential count of N60L36E1M3.

Patient was noncompliant to medication for last 2 days. Intake of junk food and other symptoms along with normal Liver Function Test (LFT) narrowed the diagnosis to acute gastroenteritis (AGE). AGE along with non-compliance resulted in an adrenal crisis in our patient. His initial blood gas analysis showed metabolic acidosis (Table 1).

**Table 1:** Haematological and biochemical investigations.

	2079/03/29	2079/03/30	2079/03/31	2079/03/32
TC	20100/mm3			
DC	N60 L36 E1 M3			
Hemoglobin	13.4 gm%			
Platelet	493000/mm3			
Na/K	124/5.8	133/5.7	137/4.3	
Urea/Creatinine	73/0.3		22/0.2	
CRP	104 mg/l			
Urine				
Acetone			Positive	Negative
Sugar			Nil	
Blood sugar				
Random	16 mg/dl			
PP				101 mg/dl
Fasting				86 mg/dl
ABG	pH:7.27 HCO3-act:11.7 mmol/l pCO2:25.6 mmHg			

The patient was managed with nebulization and NS bolus and then referred to our centre for further evaluation and management. At our centre patient was managed with IV fluids, midazolam, hydrocortisone and broad-spectrum antibiotics. The patient showed progressive improvement and was discharged on the fifth day of admission with syrup prednisolone and tablet Fludrocortisone.

## DISCUSSION

CAH is an autosomal recessive disease caused by mutations or deletions in CYP21A2, the gene encoding steroid 21-hydroxylase.^6^ Based on the data from neonatal screening, the prevalence of congenital adrenal hyperplasia (CAH) is 1 in 15,000 live births.^3,7^

The most common cause of congenital adrenal hyperplasia (CAH) worldwide, accounting for >95 per cent of cases, is a 21-hydroxylase deficiency (21-OHD).^8^ Individuals with classic 21-hydroxylase deficiency (21-OHD) have a defective conversion of 17-hydroxyprogesterone (17OHP) to 11-deoxycortisol in patients which results in decreased cortisol and aldosterone synthesis, loss of negative feedback, and therefore increased corticotropin (ACTH) secretion.^9^ The goal of treating classic CAH is to reduce the excess adrenal androgen production and replace the deficient hormones, namely cortisol and aldosterone.^10^ Proper treatment prevents both adrenal crisis and ongoing virilization. If left untreated, children with 21-hydroxylase deficiency (21-OHD) are at risk for bone age advancement, early puberty, premature epiphyseal closure, and adult short stature, from early exposure to high levels of sex hormones.^9^ The diagnosis of classic 21-hydroxylase deficiency (21-OHD) is based upon a very high serum concentration of 17-hydroxyprogesterone (17-OHP), the normal substrate for 21-hydroxylase, which is a part of routine newborn screening in developed countries.^11,12^

Unfortunately, in resource-limited settings such as our screening is not done routinely. Our case was first diagnosed with CAH at 40 days of life after he presented with features of adrenal crisis and lab results subsequently showed elevated levels of 17-hydroxyprogesterone (17-OHP). Since then the patient has been kept on prednisolone and Tablet Fludrocortisone. It is difficult to suspect CAH, especially among males because of normal-appearing genitalia at birth. Subtle findings such as hyperpigmentation of the scrotum or an enlarged phallus are frequently missed.^13^ Hence males are particularly prone to the occurrence of associated salt adrenal crisis and death.^14^

Female infants with classic 21-OHD are typically born with atypical genitalia characterized by clitoral enlargement labial fusion, and formation of a urogenital sinus and rarely, virilization may be so profound that genital atypia is unrecognized, and are assigned as males.^14^ Unlike in males it might go undiagnosed as resultant is male genitalia.^15^ As our case was non-compliant with medications for 2 days, he presented with typical symptoms of adrenal crisis such as diarrhoea, vomiting and seizures. His lab results with hyperkalemia, hyponatremia, metabolic acidosis, and hypoglycemia were consistent with the diagnosis of Adrenal crisis.^3^

The patient also had a history of intake of junk food and routine stool routine microscopy showed undigested food particles. Thus, he was subsequently diagnosed as CAH in adrenal crisis with Acute gastroenteritis. Stress doses of glucocorticoids are warranted for illnesses associated with fever, vomiting, and/or marked diarrhoea and significant trauma.^16^

Incompliance and acute stressor in the form of gastroenteritis aggravated the patient's pre-existing CAH and hence the patient presented with features of adrenal crisis. Once diagnosed the patient was treated urgently which aimed at correct of hypotension, dehydration, electrolyte and glucose abnormalities, and correction of cortisol deficiency.

The clinical spectrum of CAH ranges from the most severe to mild forms, depending upon the degree of enzyme deficiency. In settings where routine screening of CAH is not done infants, especially males, are diagnosed when they present with adrenal crisis. Timely recognition and appropriate medical management of our case have prevented the fatality. Finally, the patient party was counselled about the crucial aspect of lifelong corticosteroids and mineralocorticoids to prevent the recurrence of the adrenal crisis.

## References

[1] El-Maouche D, Arlt W, Merke DP (2017). Congenital adrenal hyperplasia.. Lancet..

[2] Munck A, Guyre PM, Holbrook NJ (1984). Physiological functions of glucocorticoids in stress and their relation to pharmacological actions.. Endocr Rev..

[3] Merke DP, Bornstein SR (2005). Congenital adrenal hyperplasia.. Lancet..

[4] Bornstein SR, Allolio B, Arlt W, Barthel A, Don-Wauchope A, Hammer GD (2016). Diagnosis and Treatment of Primary Adrenal Insufficiency: An Endocrine Society Clinical Practice Guideline.. J Clin Endocrinol Metab..

[5] Lousada LM, Mendonca BB, Bachega TASS (2021). Adrenal crisis and mortality rate in adrenal insufficiency and congenital adrenal hyperplasia.. Arch Endocrinol Metab..

[6] Speiser PW, White PC (2003). Congenital adrenal hyperplasia.. N Engl J Med..

[7] Therrell BL (2001). Newborn screening for congenital adrenal hyperplasia.. Endocrinol Metab Clin North Am..

[8] Merke DP, Bornstein SR (2005). Congenital adrenal hyperplasia.. Lancet..

[9] Yau M, Gujral J, New MI, Endotext, Feingold KR, Anawalt B, Boyce A (2000).

[10] Speiser PW (2015). Congenital Adrenal Hyperplasia.. F1000Res.

[11] Speiser PW, Arlt W, Auchus RJ (2018). Congenital Adrenal Hyperplasia Due to Steroid 21-Hydroxylase Deficiency: An Endocrine Society Clinical Practice Guideline.. J Clin Endocrinol Metab..

[12] Kaye CI, Accurso F, Committee on Genetics (2006). Newborn screening fact sheets.. Pediatrics..

[13] Gehrmann K, Engels M, Bennecke E (2019). Sexuality in Males With Congenital Adrenal Hyperplasia Resulting From 21-Hydroxylase Deficiency.. J Endocr Soc..

[14] Podgorski R, Aebisher D, Stompor M, Podgorska D, Mazur A (2018). Congenital adrenal hyperplasia: clinical symptoms and diagnostic methods.. Acta Biochim Pol..

[15] Khanal D, Mandal D, Phuyal R, Adhikari U (2020). Congenital Adrenal Hyperplasia with Salt Wasting Crisis: A Case Report.. JNMA J Nepal Med Assoc..

[16] Munck A, Guyre PM, Holbrook NJ (1984). Physiological functions of glucocorticoids in stress and their relation to pharmacological actions.. Endocr Rev..

